# The effects and costs of an anti-bullying program (KiVa) in UK primary schools: a multicenter cluster randomized controlled trial

**DOI:** 10.1017/S0033291724002666

**Published:** 2024-11

**Authors:** Lucy Bowes, Malavika Babu, Julia R. Badger, Matthew R. Broome, Rebecca Cannings-John, Suzy Clarkson, Elinor Coulman, Rhiannon Tudor Edwards, Tamsin Ford, Richard P. Hastings, Rachel Hayes, Fiona Lugg-Widger, Eleri Owen-Jones, Paul Patterson, Jeremy Segrott, Mia Sydenham, Julia Townson, Richard C. Watkins, Holly Whiteley, Margiad E. Williams, the Stand Together Team, Judy Hutchings

**Affiliations:** 1 Department of Experimental Psychology, Oxford University, Oxford, UK; 2 National Institute for Health and Care Research (NIHR) Oxford Health Biomedical Research Centre, Oxford University, Oxford, UK; 3 Centre for Trials Research, Cardiff University, Cardiff, UK; 4 School of Educational Learning and Communication Sciences, University of Warwick, Warwick, UK; 5 Institute for Mental Health, University of Birmingham, Birmingham, UK; 6 Birmingham Women's and Children's NHS Foundation Trust, Birmingham, UK; 7 Centre for Evidence-Based Early Intervention, Bangor University, Bangor, UK; 8 Department of Psychiatry, University of Cambridge, Cambridge, UK; 9 Faculty of Health and Life Sciences, Department for Public Health and Sport Sciences, University of Exeter, Exeter, UK; 10 Birmingham Women's and Children's NHS Foundation Trust, Birmingham, UK; 11 GwE (North Wales Regional School Improvement Service), Conwy, UK

**Keywords:** bullying, schools, primary education, KiVa program, randomized controlled trial, costs and cost analysis, victimization, United Kingdom, program evaluation, intervention studies

## Abstract

**Background:**

Childhood bullying is a public health priority. We evaluated the effectiveness and costs of KiVa, a whole-school anti-bullying program that targets the peer context.

**Methods:**

A two-arm pragmatic multicenter cluster randomized controlled trial with embedded economic evaluation. Schools were randomized to KiVa-intervention or usual practice (UP), stratified on school size and Free School Meals eligibility. KiVa was delivered by trained teachers across one school year. Follow-up was at 12 months post randomization. Primary outcome: student-reported bullying-victimization; secondary outcomes: self-reported bullying-perpetration, participant roles in bullying, empathy and teacher-reported Strengths and Difficulties Questionnaire. Outcomes were analyzed using multilevel linear and logistic regression models.

**Findings:**

Between 8/11/2019–12/02/2021, 118 primary schools were recruited in four trial sites, 11 111 students in primary analysis (KiVa-intervention: *n* = 5944; 49.6% female; UP: *n* = 5167, 49.0% female). At baseline, 21.6% of students reported being bullied in the UP group and 20.3% in the KiVa-intervention group, reducing to 20.7% in the UP group and 17.7% in the KiVa-intervention group at follow-up (odds ratio 0.87; 95% confidence interval 0.78 to 0.97, *p* value = 0.009). Students in the KiVa group had significantly higher empathy and reduced peer problems. We found no differences in bullying perpetration, school wellbeing, emotional or behavioral problems. A priori subgroup analyses revealed no differences in effectiveness by socioeconomic gradient, or by gender. KiVa costs £20.78 more per pupil than usual practice in the first year, and £1.65 more per pupil in subsequent years.

**Interpretation:**

The KiVa anti-bullying program is effective at reducing bullying victimization with small-moderate effects of public health importance.

**Funding:**

The study was funded by the UK National Institute for Health and Care Research (NIHR) Public Health Research program (17-92-11). Intervention costs were funded by the Rayne Foundation, GwE North Wales Regional School Improvement Service, Children's Services, Devon County Council and HSBC Global Services (UK) Ltd.

## Introduction

Bullying in childhood may be one of the most tractable risk factors for mental health problems in childhood and adolescence (Clarkson et al., 2022; Ford, Mitrofan, & Wolpert, 2014). Generally defined as a pattern of ‘unwanted, aggressive behaviour … that involves a real or perceived imbalance of power’ (Olweus, 1994), the psychiatric morbidity that may arise from childhood bullying is substantial. Population studies suggest that 25–40% of mental health problems including depression, anxiety and self-harm in young adults may be attributable to childhood bullying (Bowes, Joinson, Wolke, & Lewis, 2015; Fisher et al., 2012). Bullied children access more school health, primary care and specialist child mental health services than their counterparts (Brimblecombe et al., 2018) and experience poorer mental and physical health into adulthood (Takizawa, Maughan, & Arseneault, 2014). Bullying is also associated with school absenteeism (Brown, Clery, & Ferguson, 2011), which may impact on future educational attainment and employment prospects. Furthermore, children who bully are also at risk of harm and more likely to show later violent behavior and illicit drug use (Ttofi, Farrington, Losel, Crago, & Theodorakis, 2016). Universal whole-school interventions, promoting school-wide change are the most effective at reducing bullying (Gaffney, Farrington, & Ttofi, 2019) and likely to provide a non-stigmatizing approach to prevention. One of the most widely used bullying prevention programs across Europe is the Finnish Kiusaamisen Vastaan or ‘KiVa’ program. The program was informed by a social architecture model of bullying, which highlights the significant roles of bystanders in supporting or standing against bullying (Salmivalli, Lagerspetz, Bjorkqvist, & Osterman, 1996). In a large-scale RCT conducted in Finland between 2007–2009 across 234 schools (8166 students), KiVa significantly reduced bullying and victimization among 7 to 11-year-old students (Karna et al., 2011b) and reducing anxiety and depression (Williford et al., 2012). Since 2009, KiVa has been implemented in over 90% of Finnish public schools (approximately 2700 schools) and demonstrates year-on-year positive effects (Karna et al., 2011a). Whilst KiVa has been found to be successful in reducing victimization in trials in the Netherlands (Huitsing et al., 2020) and Italy (Nocentini & Menesini, 2016), other trials, including in Wales, found no effect (Axford et al., 2020). There are important differences between the education systems in the UK and Finland that may influence whether KiVa intervention effects will transfer. The route to becoming a teacher in the UK is more varied and children are tested more to assess their academic capabilities. Schools are typically more diverse and have greater social inequalities (Hadjar & Uusitalo, 2016). Whilst every school in the UK is required by law to have an anti-bullying policy, more guidance on effective, evidence-based anti-bullying programs is needed. In this two-arm pragmatic multicenter cluster randomized controlled trial (cRCT) with embedded economic evaluation, our primary aim was to assess the effectiveness and costs of the KiVa anti-bullying program in reducing student-reported bullying victimization. Additionally, a range of secondary outcomes were explored including student-reported bullying perpetration, participant roles in bullying and school related well-being; teacher-reported student emotional and behavioral difficulties; and teachers' self-efficacy in dealing with bullying, mental well-being, and burnout.

## Methods

A full outline of the research methodology can be found in our protocol paper (Clarkson et al., 2022).

### Study design

We conducted a two-arm pragmatic, multicenter, cluster RCT with an embedded economic evaluation. Schools and students were recruited across four areas (North Wales, Birmingham, Southeast England, and Southwest England).

Ethical approval was granted by Bangor University Psychology Research Ethics and Governance Committee (2019-16592) on 13th November 2019. The authors assert that all procedures contributing to this work comply with the ethical standards of the relevant national and institutional committees on human experimentation and with the Helsinki Declaration of 1975, as revised in 2008.

### Schools and participants

We included mainstream UK state-maintained primary schools with at least two KS (Key Stage) 2 classes for children aged 7–11 years. We excluded schools that: primarily delivered education through a language other than English or Welsh; were already implementing a recognized anti-bullying intervention program or had previously implemented KiVa; catered solely for children with Special Educational Needs; and schools without a leadership that could guarantee project participation for the year of data collection/ implementation. All students at participating schools in school years 3–5 (KS 2) were eligible for the trial. Head teachers provided opt-in consent for school-level data collection and the implementation of KiVa; parents/carers provided opt-out consent for data collection relating to their child; and teaching staff provided opt-in consent for data about themselves. Parents, carers or teachers could not withhold consent for KiVa to be delivered as part of the school's statutory personal, social education/personal, social, health and economic education (PSE/PSHE) curriculum. Children provided their written assent via e-tablets for self-reported outcomes at each data collection point. Prior to the 12-month follow-up, all parents received a letter detailing the plans to link their child's trial data to data held by the Department for Education, National Pupil Database (NPD) and in Wales, the Welsh Secure, Anonymised Information Linkage (SAIL) database. Parents had the opportunity to opt-out of this activity without it impacting on their child's continued participation in the trial.

### Randomization and masking

Schools were randomized to receive the KiVa intervention or continue with UP by the Centre for Trials Research at Cardiff University using random permuted blocks in a 1:1 ratio. Randomization was stratified by the following: study area (North Wales, Birmingham, Southeast and Southwest England); number of KS2 students (UK school years 3–6, ages 7–11) (below or above site median); and percentage of students eligible for Free School Meals (FSM) (below or above site median). Randomization of schools occurred after all schools had been recruited. Due to COVID-19 pandemic related school closures between January and March 2021 delaying the timing of child-reported baseline data collection and the need for intervention schools to be trained in KiVa before the end of academic year, 48% of schools were informed of their allocation before child-reported baseline data were collected. Most (94%) of teacher reported TSDQ data were collected before schools were informed of their allocation. Researchers collecting baseline school data were blind to the randomization process, as were students (who were the source of the primary outcome data). It was not possible to blind researchers and field workers at follow-up due to the requirement that KiVa schools make the intervention public (e.g. through posters and staff tabards). The statistician analyzing the primary and secondary outcome data and the health economists undertaking the economic analysis remained blinded.

### Patient and public involvement

Two advisory groups were established in North Wales, one with teachers and head teachers, and one with six children from three different primary schools. Groups met twice at the start of the project and reviewed and advised on data collection methods, participant information and consent forms. The groups reconvened after the COVID-19 pandemic in 2023 to advise on the output and dissemination activities.

### Procedures

Baseline survey data were collected from students in school years 3–5 between 20^th^ April–30^th^ June 2021. Child questionnaires were administered via e-tablets with researchers reading the questions aloud to the whole class. All questions included the option ‘I'd rather not say’. Teachers completed questionnaires either online or with paper and pen. The KiVa anti-bullying intervention was implemented in the academic year which ran from September 2021 to July 2022, and survey outcomes were measured from 26^th^ April–15^th^ July 2022.

### Intervention

KiVa is based on research demonstrating that ‘bystanders’ – children who are present during bullying but not actively involved – can contribute to the maintenance of bullying by assisting or reinforcing the perpetrator's behavior, giving the bully a position of power (Salmivalli et al., 1996). Defending the victim, on the contrary, can help to make bullying an unsuccessful strategy for attaining high social status. By influencing the behavior and norms of all students, the social rewards gained by perpetrators are reduced and, consequently, reduces their motivation to bully. The KiVa program has two distinct components; universal actions that target the whole school and indicated actions for tackling specific cases of bullying. Universal actions at the class and school level help students to recognize bullying, providing them with safe ways of responding to incidents, and teaching them to empathize with, and support, victims. This includes structured lessons, delivered by teachers. The manualized curriculum targets students in school Years 3 and 4 (aged 7–9) (Unit 1), and Years 5 and 6 (aged 9–11) (Unit 2). Although the lesson plans target KS2 classes, the program is introduced to all staff, parents and students and is visible across the school. Each unit contains ten structured 90-minute lessons, typically delivered fortnightly as twenty 45-minute lessons throughout the school year by class teachers. The curriculum encourages student engagement via oral presentations, role-play, videos, group work and whole class activities. Online games, that support lessons can be played at home or in school. Other universal actions include posters for school corridors, high-visibility vests for break-time supervisors to highlight the presence of supervision, and a parent's guide. A trained KiVa team (2 to 3 staff members) address confirmed bullying incidents with children involved using the structured and scripted indicated actions, with individual follow-up meetings 1–2 weeks later to ensure that the situation has improved. Two members of the teaching/management team from each intervention school attended a local two-day training course delivered in June 2021 by accredited KiVa trainers at each site and then led school-wide implementation. Due to ongoing disruption from the COVID-19 pandemic, some training was delivered in an online format.

The intervention delivery was over one academic year. However, the program is designed to be embedded into ongoing school practice.

### Comparison

The mandatory PSE/PSHE curricula in Wales and England aim to develop students' pro-social values and attitudes and empower participation in school and community life as responsible citizens. Comparison schools continued to use existing methods to cover this curriculum, and to address bullying. UP schools continued with their standard practice and were free to implement any other programs or strategies they wished to in their school. At baseline, all schools reported which existing policies and practices they used to prevent and deal with bullying. At the follow-up point all schools were asked if there had been any changes to their policies or practices during the trial period.

### Outcomes

#### Primary outcome

The primary study outcome was self-reported bullying victimization, measured by student responses on the Olweus Bully/Victim Questionnaire (OBVQ) (Olweus, 1996). The OBVQ measures different forms of bullying, including verbal, physical, relational and cyber-bullying. The global item: ‘How often have you been bullied at school in the last couple of months?’ was used to measure victimization. Students respond on a five-point scale (0 = ”not at all”, 1 = ”once or twice”, 2 = ”2 or 3 times a month”, 3 = ”about once a week”, 4 = ”several times a week”). The response was dichotomized with those scoring 2 to 4 classified as victimized and those scoring 0 to 1 as not victimized (20)

#### Secondary outcomes

- Student-reported outcomes:
  1. The OBVQ global item: ‘How often have you bullied others at school in the last few months?’ measures bullying perpetration. Students' responses, using the same five-point scale reported above were analyzed continuously and dichotomized, with scores of 2 to 4 classified as perpetration (Solberg, 2003).
  2. Subjective student wellbeing in school was measured using the ‘How I feel about my school’ (HIFAMS) (Allen et al., 2018). The survey is comprised of seven items which asks students how they feel about various aspects of school life, for example how they feel when completing their schoolwork or when they are in the playground.
  3. Student empathy was measured using the Empathy Toward Victim Scale (Pöyhönen, Juvonen, & Salmivalli, 2010). The seven-item measure asked respondents to rate their level of empathy towards victims on a Likert scale anchored from ‘never’ (0) to ‘always’ (3).
  4. Roles in bullying situations was measured using the ‘Participant Role Questionnaire’ (PRQ) (Salmivalli & Voeten, 2004), designed to identify the different roles that peers play in bullying situations. The questionnaire identifies five different bullying roles; bully, assistant, reinforcer, defender and outsider. We were particularly interested in the role of defender, given that KiVa is intended to raise awareness among children about the importance of everyone taking a stand against bullying. The respondent rates how often they behave in the ways described for each role on a three-point scale (Never, Sometimes, Often).
- Teacher-reported student outcomes (measured through teacher survey reports for each student):
  1. Teacher-reported Strengths and Difficulties Questionnaire (TSDQ) (R., 2001) was reported at baseline and follow-up. The TSDQ measure provides a Total Difficulties score from 20 behavioral and emotional problems items as well as a pro-social behavior score from five items. Subscales include a hyperactivity score, emotional score, conduct problems score and peer problems score.
  2. We intended to collect data from the Absence and Exclusion, and KS2 attainment datasets for English pupils from the National Student Database (NPD) and from the Welsh Government SAIL database for students in both trial arms. However, due to COVID 19 pandemic-related delays in obtaining data, these data are not currently available. For a full list of tertiary measures (not included in this paper), see study protocol (Clarkson et al., 2022).
- Staff-level outcomes.
  1. Adapted version of the 5-item Challenging Behavior Self-Efficacy Scale, designed as a measure of teacher self-efficacy related to challenging behaviors and adapted to specifically
  2. to bullying behaviors (Hastings & Brown, 2002).
  3. Warwick-Edinburgh Mental Wellbeing Scale (Tennant et al., 2007). This 14-item positively worded scale measures adult mental wellbeing.
  4. Maslach Burnout Inventory-Educator

Survey (MBI-ES), a 22-item psychological inventory measuring emotional exhaustion, depersonalization, and personal accomplishment (Maslach, Jackson, & Schwab, 1996).

### Process evaluation

The methods for the process evaluation are reported in our protocol paper (Clarkson et al., 2022) and the results will be in a separate publication.

### Economic evaluation

In line with guidance for evaluating complex public health interventions (Skivington et al., 2021), a broad economic evaluation using cost-consequence analysis (CCA), cost-effectiveness analysis (CEA), and cost-utility analysis (CUA) frameworks explored the cost-effectiveness of the KiVa intervention compared to UP. An education-sector perspective (Edwards & McIntosh, 2019) was taken to evaluate incremental effects at 12 months after randomization. These results are reported separately.

Cost analyses from an education-sector perspective focused on two out of three components of the KiVa program; (i) class-level delivery of KiVa lessons and, (ii) whole-school activities to promote KiVa to the school population. KiVa cost components were categorized into recurrent and non-recurrent costs and unit costs were derived using appropriate UK estimates for the trial year (2021/2022) (Appendix X, Table 1). Resource use and cost data for KiVa were collated from the Children's Early Intervention Trust (CEIT) charity that holds the license for KiVa dissemination in the UK and process evaluation school checklists. Resource use data for UP were collected via PSE/PSHE school checklists. Cost calculations for usual practice considered the school-reported overlap between the KiVa and PSE/PSHE curriculums. Where there were missing data at the school-level mean imputation was used unless there was evidence that these costs had not been incurred. Discounting was not applied.

**Table 1.** Baseline characteristics for students and teachers (Data sources: Pupil, TSD and teacher questionnaire)

	KiVa	Usual practice
Number of schools	59	59
Cluster size (Students per school) Median (IQR)	81 (45, 114)	82 (40, 152)
Number of students	5944	5167
Trial site		
North Wales	1556 (26.1%)	1148 (22.2%)
Southwest England	1225 (20.6%)	1106 (21.4%)
Southeast England	1168 (19.7%)	1161 (22.5%)
Birmingham	1995 (33.6%)	1752 (33.9%)
Student reported characteristics		
Females	2905/5933 (49.0%)	2560/5166 (49.6%)
Age (in years)^a^	*N* = 5937	*N* = 5165
Mean (s.d.)	8.7 (0.97)	8.7 (0.94)
OBVQ – Self-reported bullying victimization^b^		
Yes	1028/5074 (20.3%)	955/4418 (21.6%)
No	4046/5074 (79.7%)	3463/4418 (78.4%)
OBVQ – Self-reported bullying perpetration^c^		
Yes	209 (4.0%)	168 (3.7%)
No	5012 (96.0%)	4395 (96.3%)
HIFAMS^d^ Mean (s.d.)	11.04 (2.3)	11.01 (2.4)
ETV^e^ Mean (s.d.)	11.68 (5.0)	11.74 (5.0)
PRQ		
Bully scale		
Never (score = 0)	4541 (86.1%)	4017 (87.3%)
Sometimes/Always (score>0)	735 (13.9%)	586 (12.7%)
Assistance scale		
Never (score = 0)	4340 (81.4%)	3729 (81.5%)
Sometimes/Always (score>0)	995 (18.7%)	846 (18.5%)
Reinforcer scale		
Never (score = 0)	1026 (19.1%)	947 (20.3%)
Sometimes/Always (score>0)	4342 (80.9%)	3722 (79.7%)
Defender scale		
Never (score = 0)	108 (2.1)	116 (2.5)
Sometimes/Always (score>0)	5173 (97.9)	4515 (97.5)
Outsider scale		
Never (score = 0)	274 (5.8%)	262 (6.4%)
Sometimes/Always (score>0)	4432 (94.2%)	3864 (93.7%)
Teacher reported student based		
Number of students	6159^f^	5412^f^
TSDQ^g^ Median (IQR)		
Total difficulties score^h^(range 0–40)	4 (1, 9)	4 (1, 9)
Prosocial behavior score (range 0–10)	9 (6, 10)	9 (6, 10)
Hyperactivity score (range 0–10)	2 (0, 5)	2 (0, 5)
Emotional problems score (range 0–10)	1 (0, 2.5)	1 (0, 2)
Conduct problems score (range 0–10)	0 (0, 1)	0 (0, 1)
Peer problems score (range 0–10)	0 (0, 2)	0 (0, 2)
Teacher reported characteristics		
Number of teachers	228	223
Job role *n* (%)		
Headteacher/Executive headteacher	3 (1.3%)	0 (0.0%)
Deputy or assistant Headteacher/Senior Leadership teacher/Key stage and subject specific leads	31 (13.7%)	30 (13.6%)
Teacher or associate teacher	116 (51.3%)	116 (52.7%)
Higher level teaching assistant or trainee teacher	4 (1.8%)	12 (5.5%)
Teaching, classroom or learning support assistants	55 (24.3%)	53 (24.1%)
Special educational needs coordinator/Pastoral lead/Behavior Mentor/designated safeguarding leader/Wellbeing	11 (4.9%)	6 (2.7%)
1:1 Supports/Learning mentors/Additional learning needs	6 (2.7%)	3 (1.4%)
Duration in role *n* (%)		
Up to 10 years	168 (74.3%)	178 (80.9%)
11–20 years	45 (19.9%)	35 (15.9%)
Greater than 20 years	13 (5.8%)	7 (3.2%)
MBI-GS Median (IQR)		
Emotional exhaustion^i^	15 (9, 22)	16 (10, 22)
Cynicism^i^	8 (2, 14)	7 (3, 13)
Professional efficacy^j^	28 (24, 31)	28 (24.5, 31)
WEMWBS^l^ Mean (s.d.)	49.0 (8.2)	49.2 (8.0)
CBSES^k^ Median (IQR)	27 (24, 30)	27 (23, 29)

UP, Usual practice; OBVQ, Olweus Bully/Victim Questionnaire; HIFAMS, How I Feel About My School; ETV, Empathy Toward Victim Scale; PRQ, Participant Role Questionnaire; TSDQ, Teacher Strength and Difficulties Questionnaire; MBI-GS, Maslach Burn-Out Inventory General Survey; WEMWBS, Warwick-Edinburgh Mental Well-being Scale; CBSES, Challenging Behavior Self-Efficacy Scale.

Data are *n* (%), mean (s.d.) or median (IQR).

a Five children self-reported being six years of age despite being in school years 3–5.

b Data were missing for 870 (KiVa) and 749 (UP) students. Bullying victimization defined as ‘How often have you been bullied at school in the past couple of months?’ = 2 or 3 times a month/About once a week/Several times a week.

c Data were missing for 723 (KiVa) and 604 (UP) students. Bullying perpetration defined as ‘How often have you taken part in bullying another pupil(s) at school in the past couple of months?’ = 2 or 3 times a month/About once a week/Several times a week.

d Data were missing for 269 (KiVa) and 211 (UP) students. HIFAMS scores range from 0 to 14 – higher scores reflect greater happiness.

e Data were missing for 338 (KiVa) and 310 (UP) students. ETV scores range from 0 to 21 – higher scores indicate greater empathy for the victim.

f Teachers reported TSDQs for a higher number of students compared to the pupils self-reported questionnaires.

g Data were missing for between 2 and 5 students. For all subscales, higher scores indicate more strength or difficulty.

h Total of all subscales excluding Prosocial behavior subscale.

i Data were missing for between 10 and 15 teachers. Scores range from 0 to 30.

j Data were missing for 14 (KiVa) and 11 (UP) teachers. Scores range from 0 to 36.

k Data were missing for 8 (KiVa) and 9 (UP) teachers. CBSES scores range from 5 to 35.

l Data were missing for 7 (KiVa) and 6 (UP) teachers. WEMWBS scores range from 14 to 70.

### Safety and adverse events

There were no anticipated risks to participants or schools.

### Statistical analyses

Our sample calculation was based on previous research (reduction from 18% to 14% in rates of victimization) (Skivington et al., 2021), and a similar baseline victimization rate of 18% from a small UK based pre-post study (Edwards & McIntosh, 2019). Assuming 111 students in Years 3 to 5, an intracluster correlation coefficient (ICC) of 0.02, and allowing for one school dropout per arm, 10% student dropout due to either opt-out or loss to follow-up, an 18% rate of victimization, and a relative reduction of 22%, a trial involving 116 schools (58 per arm) would provide 90% power at a 5% significance level (a total of 12 828 students).

All analyses were intention to treat without imputation (a complete case analysis restricting to pupils with responses at both baseline and follow-up), with outcomes compared between KiVa and UP groups using three-level regression models (allowing for clustering between students within school, and between schools within sites). Analyses controlled for school level stratification variables (school size, proportion of students eligible for FSM), key student characteristics (age, sex) as well as baseline outcome measures (where collected).

For binary outcomes a logistic model was used, and the result presented as adjusted odds ratios (ORs) comparing the odds of an event in KiVa schools compared with UP schools. For continuous outcomes, we fitted a linear-regression model and presented results as difference in adjusted means (KiVa minus UP). Multilevel ordinal logistic regression model was used to compare TSDQ scales. Due to skewness in the TSDQ scales, data were categorized according to the clinical cut-offs (normal, borderline, abnormal). Box-cox transformations were applied to skewed data when necessary and Glass's delta standardized effect size calculated as the difference in means (KiVa – UP) divided by the standard deviation of the UP group. Between-group comparisons are presented with two-sided 95% confidence intervals (CI). Pre-specified exploratory sub-group analyses investigated the effect of the intervention by student gender and school-level percentage of students eligible for FSM status. Three additional post-hoc subgroups were performed: Pupils self-reported age (6–7, 8, 9, 10/11 years of age), whether the school previously recruited before COVID-19 pandemic for the Stand Together trial, and the timing of baseline data collection. For all subgroup analyses, an interaction term was added to the main comparison model between the study arm and each variable and results presented using 95% CIs.

The impact of missing outcome data on the trial conclusions, missing mechanisms were explored, and appropriate imputation methods applied via sensitivity analyses.

The effect of KiVa lesson dosage on the primary outcome was explored in a sensitivity analysis and estimated in a way that preserves randomization using complier-average causal effect estimates, using a two-stage least squares instrumental variable regression model (Dunn, Maracy, & Tomenson, 2005).

The secondary outcome of the OBVQ bullying victimization and perpetrator score was originally omitted from the SAP but is included as a post-hoc analysis (ordinal logistic regression).

In exploratory analyses, mediation analyses were used to determine whether the effectiveness of the intervention on bullying victimization occurred through change in affective empathy and secondly with change self-efficacy in defending. The mediation analysis was parameterized as a 2-1-1 model where the independent variable (intervention) is at the school level, while the mediator (affective empathy or self-efficacy) and the outcome (bullying victimization) are at the pupil level. Both models adjusted for pupil-level characteristics (age, gender), school level characteristics (FSM, KS2) along with corresponding baseline measures.

Analyses were done by two statisticians (MB and RCJ) using IBM SPSS Statistics version 20.0 (IBM Corporation, Armonk, NY, USA), and Stata® version 17 (StataCorp LP, College Station, TX, USA).

An independent steering committee oversaw the study. The trial is registered at the ISRCTN 12300853.

## Results

**Figure 1.**
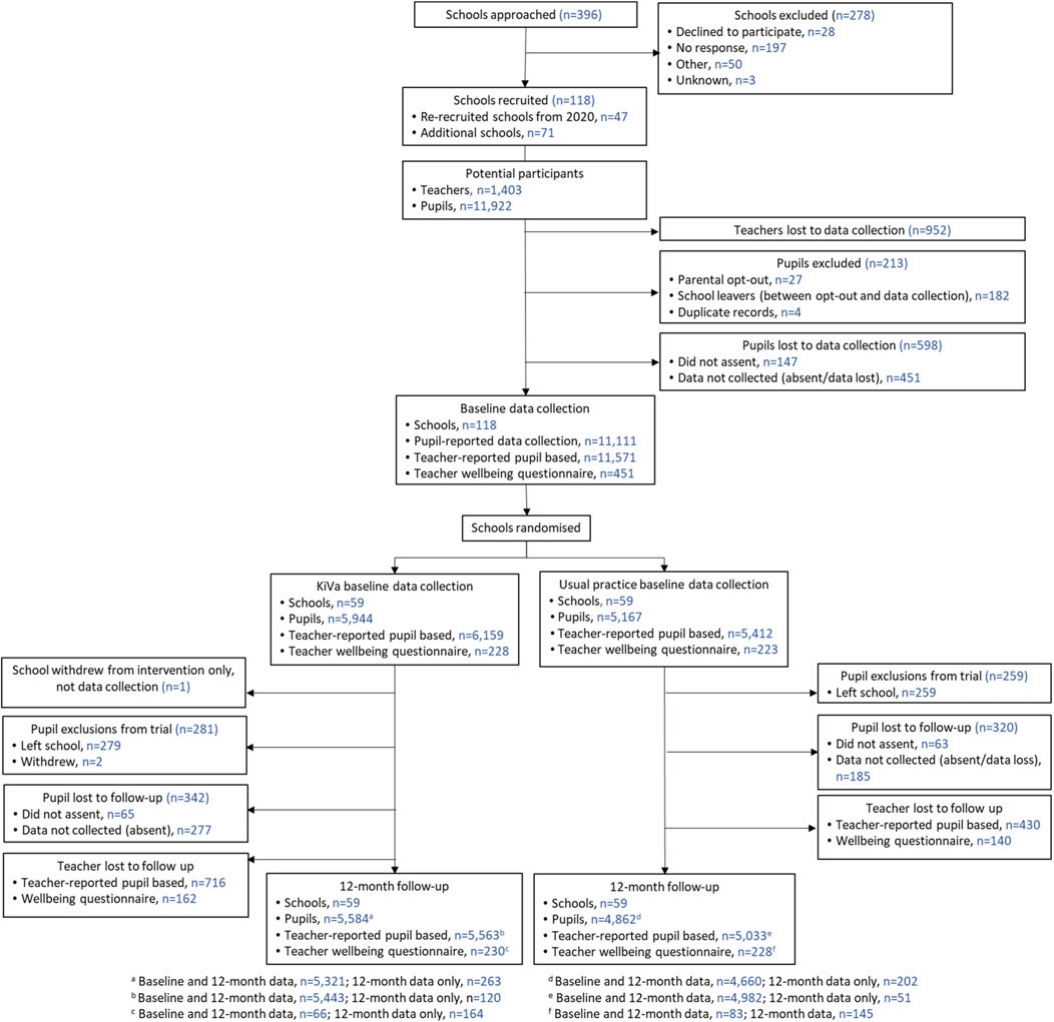
The CONSORT Flow Diagram. *Of the 118 schools recruited to the study, 47 had previously completed baseline data for the Stand Together Trial prior to the COVID 19 pandemic and were re-recruited when the study restarted.

See Fig. 1 for the CONSORT Flow Diagram. Between 8^th^ November 2019 and 12^th^ February 2021, 118 schools were recruited from 396 schools approached across four trial sites. From a potential of 11 922 students, 11 111 (93.2%) students completed a baseline questionnaire. Twelve month-follow up data was collected from 9981 (90.1%) students, 5321 (90.0%) students in the KiVa schools compared to 4660 (90.2%) in UP schools. In addition, a total of 263 (KiVa) and 202 (UP) questionnaires were completed by students who had not completed a baseline questionnaire. Baseline student and teacher characteristics were well balanced across trial arms (Table 1). Forty-nine percent of students were female and on average aged 8.7 years.

The total number of students available for the complete case analysis (baseline and 12-month follow-up) was 9981 (Table 2). At baseline, 20.3% of students in KiVa schools and 21.6% of students in UP schools experienced bullying victimization. After 12-month follow-up, the bullying victimization rate was reduced to 17.7% in the KiVa arm compared to 20.7% in the UP arm. The adjusted odds ratio indicated that the students from schools randomized to receive KiVa had a 13% lower odds of reporting bullying victimization compared to students in schools randomized to continue with UP (Table 2) (odds ratio (OR): 0.87, 95% CI 0.78–0.97, *p* value = 0.009). Pre-specified sensitivity analyses were performed to investigate the effect of dosage (at least seven out of the 10 annual lessons delivered over a full school year to each class) on intervention effect, and imputation for missing data. The intervention effect was consistent across all sensitivity analyses (appendix, Table S1.1). No differential effects were found for any primary outcome in subgroup analyses (appendix, Table S2.1). The additional post-hoc analysis on the maximum OBVQ bullying victimization and perpetrator score, supported the primary analysis (Table 3).

**Table 2.** Primary and secondary outcomes for students (Data sources: Pupil and TSD questionnaire)

	12-month follow-up			
	KiVa	Usual practice	Adjusted^a^ intervention effect (95% CI)	*p* value
Total students	*N* = 5584	*N* = 4862		
Primary outcome				
OBVQ – Bullying victimization				
Self-reporting bullying victimization	885 (17.7%)	873 (20.7%)	0.87^b^ (0.78–0.97)	0.009
Not self -reporting bullying victimization	4109 (82.3%)	3347 (79.3%)	..	..
Secondary outcomes				
OBVQ – Bullying perpetration				
Self-reporting bullying perpetration	141 (2.8%)	149 (3.4%)	0.90^b^ (0.80–1.01)	0.08
Not self -reporting bullying perpetration	4954 (97.2%)	4238 (96.6%)	..	..
HIFAMS Mean (s.d.)	10.3 (2.6)	10.3 (2.6)	0.02^c^ (−0.08 to 0.11)	0.68
ETVS Mean (s.d.)	11.0 (4.9)	10.8 (4.9)	0.45^c^ (0.20–0.69)	<0.001
PRQ – Defender Scale				
Sometimes/Always (score>0)	4784 (97.0)	4190 (97.1)	0.76^b^ (0.59–0.97)	0.030
Never (score = 0)	148 (3.0)	127 (2.9)	..	..
Teacher reported student based				
Total students	*N* = 5563	*N* = 5033		
TSDQ				
Total score				
Normal	4918 (88.4%)	4426 (88.0%)		
Borderline	279 (5.0%)	273 (5.4%)	reference	
Abnormal	364 (6.6%)	332 (6.6%)	1.01^b^ (0.78–1.30)	0.96
Prosocial score				
Normal	4358 (78.4%)	3835 (76.2%)		
Borderline	386 (6.9%)	385 (7.7%)	reference	
Abnormal	817 (14.7%)	811 (16.1%)	0.93^b^ (0.77–1.11)	0.40
Hyperactivity score				
Normal	3893 (70.0%)	3502 (69.6%)		
Borderline	384 (6.9%)	341 (6.8%)	reference	
Abnormal	1284 (23.1%)	1187 (23.6%)	1.02^b^ (0.91–1.14)	0.73
Emotional score				
Normal	4147 (74.6%)	3680 (73.2%)		
Borderline	470 (8.4%)	445 (8.8%)	reference	
Abnormal	944 (17.0%)	906 (18.0%)	1.01^b^ (0.83–1.23)	0.93
Conduct problems score				
Normal	5219 (93.9%)	4696 (93.3%)		
Borderline	141 (2.5%)	149 (3.0%)	reference	
Abnormal	201 (3.6%)	186 (3.7%)	0.87^b^ (0.66–1.16)	0.34
Peer problem score				
Normal	4775 (85.9%)	4242 (84.3%)		
Borderline	336 (6.0%)	357 (7.1%)	reference	
Abnormal	450 (8.1%)	432 (8.6%)	0.85^b^ (0.75–0.96)	0.011

OBVQ, Olweus Bully/Victim Questionnaire; HIFAMS, How I Feel About My School; ETV, Empathy Toward Victim Scale; PRQ, Participant Role Questionnaire; TSDQ, Teacher Strength and Difficulties Questionnaire.

Data are *n* (%). Mean (s.d.) or median (IQR).

a Adjusted for free school meals, KS2 school size, corresponding baseline measure, age and gender of pupil, and clustered within 118 schools within 4 recruitment sites.

b Adjusted odds ratio for KiVa *v.* usual practice.

c Adjusted difference in means for KiVa minus usual practice.

**Table 3.** OBVQ maximum self-reported victimization and perpetration scores – post-hoc analysis

			12-month follow-up			
	KiVa	Usual Practice	KiVa	Usual practice	Adjusted^a^ intervention effect (95% CI)	*p* value
Total students	5944	5167	*N* = 5584	*N* = 4862		
Bullying victimization					0.80 (0.71–0.90)	<0.001
1 – Not at all	2818 (47.4)	2382 (46.1)	1314 (23.8)	946 (19.7)		
2 – Once or twice	1.228 (20.7)	1081 (20.9)	2113 (38.2)	1810 (37.7)		
3–2 or 3 times a month	389 (6.5)	376 (7.3)	727 (13.1)	707 (14.7)		
4 – About once a week	259 (4.6)	264 (5.1)	576 (10.4)	543 (11.3)		
5 – Several times a week	380 (6.4)	315 (6.1)	803 (14.5)	795 (16.6)		
Bullying perpetration					0.76 (0.69–0.84)	<0.001
1 – Not at all	4387 (73.8)	3.830 (74.1)	3470 (63.3)	2756 (57.8)		
2 – Once or twice	625 (10.5)	565 (10.9)	1546 (28.2)	1511 (31.7)		
3–2 or 3 times a month	93 (1.6)	81 (1.6)	218 (4.0)	230 (4.8)		
4 – About once a week	58 (0.98)	47 (0.91)	104 (1.9)	130 (2.7)		
5 – Several times a week	58 (0.98)	40 (0.8)	146 (2.7)	144 (3.0)		

Data are *n* (%).OBVQ = Olweus Bully/Victim Questionnaire.

a Adjusted for free school meals, KS2 school size, corresponding baseline measure, age and gender of pupil, and clustered within 118 schools within 4 recruitment sites.

Some secondary outcomes suggested positive impacts of the KiVa intervention (Table 2). Students in schools randomized to KiVa had increased empathy towards victims compared to students in the UP arm (adjusted mean difference: 0.45, 95% CI 0.20–0.69), however they reported significantly less self-efficacy in defending other children from being bullied compared to students in the UP arm (OR: 0.76, 95% CI 0.59–0.97). We found no evidence of a difference in school-related happiness (HIFAMS) or self-reported bullying perpetration. Students in the KiVa arm had 15% lower odds of having teacher-reported TSDQ peer problems as compared to students in the UP arm (OR: 0.85, 95% CI 0.75–0.96). Follow-up exploratory analysis suggested that this effect was driven by a reduction in teacher-reported peer victimization (see Table S3.2). We found no evidence of trial arm differences in the other TSDQ subdomains (including emotional problems, conduct problems, hyperactivity and prosocial behavior). We found no evidence of intervention effects on teacher self-efficacy in dealing with bullying, wellbeing, or burnout (Table S3.1).

Table S4.1 presents the direct, indirect and total effect of intervention and corresponding mediator on bullying victimization. These models indicated that as compared to usual practice, 22% effect of KiVa is mediated through change in affective empathy whereas 12.5% of this effect mediated through change in self-efficacy in defending.

### Economic evaluation

Full economic evaluation results will be reported in a separate paper. The main cost components of the KiVa intervention were training costs and staff-time spent delivering the KiVa curriculum (Appendix 5, Table S5.1). Mean total education sector-related costs of the KiVa intervention were £38.18 (s.d. £11.36) per pupil in the first year of KiVa set-up and implementation (i.e. non-recurrent and recurrent costs). Means costs were £19.05 (s.d. £3.94) per pupil for recurrent costs only. An average 40% overlap between the KiVa and PSE/PSHE curriculums was reported by intervention schools. When considering the mean cost of delivering 40% of the PSE/PSHE curriculum (£17.40 (s.d. £6.74) per pupil), the cost of KiVa compared to usual practice is estimated to be £20.78 more per pupil in the first year of KiVa set-up and implementation and £1.65 more per pupil when only recurrent costs are considered. Further details of resource use and costs are reported in the appendix.

## Discussion

The results of the study showed that the KiVa intervention reduced bullying victimization among students from KiVa schools reporting a 13% reduction in the odds of victimization compared with students from schools continuing with usual practice. The reduction in odds of bullying victimization observed in our trial (13%) was lower than reported in other studies (Finland – 30% (Karna et al., 2011b); Netherlands 43.4% (Huitsing et al., 2020), Italy 50.9% (Nocentini & Menesini, 2016)). This may reflect the significant challenges in implementing a whole-school intervention in the context of the COVID-19 global pandemic, positive effects of UP, or both. It is unclear whether a 13% reduction in bullying could be considered meaningful at the school level. The concept of identifying ‘clinically meaningful’ reductions in symptoms is common in clinical psychology but is yet to be addressed in anti-bullying interventions. (Carey, Ridler, Ford, & Stringaris, 2023).

In addition to reducing bullying victimization, students in the intervention group demonstrated increased empathy. Indeed, 22% effect of the KiVa was found to be mediated through change in affective empathy. These positive findings further support the efficacy of the KiVa program in improving the overall social dynamics within schools. Students in the intervention group had fewer reported peer relationship problems, however this effect was no longer significant when adjusting for multiple testing. We also observed some small, unexpected effects on student reported self-efficacy in defending victims of bullying, which decreased in the intervention group. These findings should be interpreted with caution and require replication in independent samples. It is also important to note that no significant differences were observed in teacher-reported emotional or conduct problems, or child-reported well-being. Whilst the original evaluation of KiVa in Finland observed a significant reduction of symptoms of anxiety (but not depression) (Williford et al., 2012), both the Dutch and Italian KiVa evaluations found small, largely non-significant effects on internalizing symptoms (Carey et al., 2023; Huitsing et al., 2020; Nocentini & Menesini, 2016). These results suggest that while the KiVa program may reduce bullying, effects may not extend to mental health outcomes.

Our exploratory findings also indicate that the effectiveness of the KiVa program may be consistent across different demographic groups, including those from more socioeconomically deprived settings and by gender. This suggests that the intervention may have the potential to benefit students from diverse backgrounds and is not limited to specific subgroups – an important finding when attempting to ensure equitable access to effective anti-bullying interventions. Furthermore, the economic evaluation suggests that the KiVa program is a low-cost intervention compared to many other UK school-based interventions. This is a significant finding and suggest that implementing whole-school approaches like KiVa is a feasible and efficient strategy for reducing bullying in schools.

Our study had several strengths. This is the largest randomized controlled trial in the world to date of the KiVa anti-bullying programme and included a broad and diverse range of schools and students. We included an embedded economic evaluation that collected data on the wider costs of bullying to schools and families. School and student participation were high. All analyses were pre-registered and were performed blind by statisticians at the Centre for Trials Research, a UKCRC Registered Trials Unit.

Limitations of our trial include our reliance on self-reported bullying victimization and perpetration, which may inflate response bias. Whilst peer-nomination is often considered the gold-standard method of measuring bullying, this is not acceptable to many schools in the UK, nor does it typically allow for measurement of bullying outside of the classroom context. It also does not capture student perceived threat. Given that teachers also reported fewer peer relationship difficulties in the intervention arm, we can be confident that our findings support our student-reported outcomes. Secondly, some schools were informed of their assignment to the intervention before student reported baseline data were collected due to the impact of COVID-19 pandemic related school closures on our data collection timetable and the need for schools to train staff and plan for the next academic year. We mitigated the impact of this by asking teachers not to inform students or research staff of their assignment, and by performing sensitivity analyses to check that this did not impact on our findings (see Supplementary Table S2.1). Additionally, some schools were recruited to our trial in 2019, prior to the COVID-19 pandemic. For these schools, baseline data collection needed to be repeated. We mitigated any impact of this repeated data collection by performing additional sensitivity analyses to ensure that intervention effects were similar for these schools (see Supplementary Table S2.1). Finally, we collected follow-up data before the end of the school year whilst pupils remained in the same classes when schools had delivered the KiVa intervention for less than one academic year. Complex, whole-school interventions typically take time to bed in, which may have contributed to the lower than predicted intervention effect sizes observed. In support of this, we found that intervention schools that had delivered more of the KiVa lessons showed a greater reduction in bullying. It is plausible that the effects of the intervention may increase as the program gets embedded within the broader school system – as found in an RCT in the Netherlands which found greater effect sizes after two years of program implementation (Huitsing et al., 2020), as well as the broader rollout of the KiVa program in Finland (Karna et al., 2011a).

Our economic evaluation compared costs of KiVa training and delivery including whole-school activities against the mean cost of delivering 40% of the PSE/PSHE curriculum in UP schools. However, it is a mandatory requirement for all schools in the UK to have anti-bullying policy and practices in place, and we were unable to cost any additional school time focused specifically on bullying in UP schools, so these costs are likely to be an underestimate of the true costs of tackling bullying in UK schools. Thus, it is probable that the relative additional costs of delivering KiVa are even smaller than reported here.

Our study was conducted during the COVID-19 pandemic, when schools were experiencing increased pressure, closures, staff and student absences and other COVID-19 restrictions. This impacted all elements of implementation, from teacher training through to lesson delivery and data collection, primarily through staff and student absences. Given this unusual context, it is notable that only one school dropped out of the intervention (but remained in the data collection), and that our data return rates were high.

## Supporting information

Bowes et al. supplementary material Bowes et al. supplementary material

## Data Availability

De-identified patient data from this study along with a data dictionary can be made available after a signed data access agreement upon reasonable request to the principal investigators of the trial (j.hutchings@bangor.ac.uk and lucy.bowes@psy.ox.ac.uk).

## Supplementary material

The supplementary material for this article can be found at https://doi.org/10.1017/S0033291724002666.
