# Supplementary material for: The effects and costs of an anti-bullying program (KiVa) in UK primary schools: a multicenter cluster randomized controlled trial
Source: Psychol Med. 2024 Nov 12;54(15):4362–73. doi: 10.1017/S0033291724002666 (PMC11650158; doi:10.1017/S0033291724002666)
Supplement: Bowes et al. supplementary material [file S0033291724002666sup001.docx]

**Appendix**

## **Supplementary material to KiVa trial report**

### **Sensitivity analyses for primary outcome**

Pre-specified sensitivity analyses were performed using (1) complier average causal effect (CACE) modelling to investigate the effect of dosage on intervention effect and (2) imputation for missing data.

#### **CACE**

The main dosage metric of KiVa was defined as the number of class lessons delivered with the expectation that at least seven out of the ten annual lessons were delivered to each class. When the effect of fidelity was examined on the intervention effect, the number of lessons delivered during the Autumn and Spring terms combined were examined as a proportion of the number of lessons due to have been delivered over the first two terms. The reason for this was the majority of KiVa delivery had been completed by the end of the Spring term and the denominator (total number of lessons due to be delivered) was adjusted to reflect the number of lessons that a school would have been expected to have delivered up to that point. From 59 schools randomised to receive KiVa, only 38 schools returned checklists in the Autumn and/or Spring term. Of these, 20 provided evidence of fidelity to KiVa (with 70% or more of the expected lessons, delivered the first two terms) and 18 did not. Table S1.1 presents the results demonstrating a significant effect (regression coefficient: 0·67, 95% CI: 0·47, 0·95, p value=0·026) when adjusting for dosage on the primary outcome.

#### **Missing data – imputation**

Multiple imputation by chained equations (MICE) were used to account for missing data where data were expected to be missing at random (MAR). Imputation was performed for missing data in the primary outcome at baseline and 12-month and final analysis based on 11,523 observations. The imputation models included all covariates, trial arm, and the outcome of interest. Fifteen imputed datasets were generated, in line with guidance suggesting that the number of imputations should at least be equal to the proportion of missing data^[[1]](#footnote-1)^. The imputed three-level logistic regression models were adjusted for its corresponding baseline measure, FSM, KS2 size, age, and gender of pupil. These models indicated that the KiVa arm had around 13% lower odds of lower odds of having bullying victimization as compared to usual practice arm (Table S1·1). The model is statistically significant at 5% level.

#### **Missing data – Best/Worst-case scenarios**

All best/worst-case scenarios consistently showed that the KiVa arm had around 13% lower odds of having bullying victimisation as compared to usual practice arm (Table S1·1). All scenarios are statistically significant at 5% level.

**Table S1.1: Primary outcome: Students self-reporting bullying victimisation, n (%) on student self-reporting bullying victimisation at 12 months follow-up**

| **Analysis** | **N** | **Adjusted^a^ OR (95% CI)** | **p-value** |
| --- | --- | --- | --- |
| **Complete case^b^** | 7,678 |  |  |
| Usual practice |  | Reference |  |
| KiVa |  | 0·87 (0·78, 0·97) | 0·009 |
| **CACE^c^** | 6,482 |  |  |
| Usual practice |  |  |  |
| KiVa |  | 0·67 (0·47, 0·95) | 0·026 |
| **Imputed** | *11,523* |  |  |
| Usual practice |  | Reference |  |
| KiVa |  | 0·84 (0·72, 0·97) | 0·017 |
| **Best/Worst Case Scenario I^d^**  *(Both Baseline and Follow-up considered as experiencing no bullying victimisation)* | *11,523* |  |  |
| Usual practice |  | Reference |  |
| KiVa |  | 0·87 (0·79, 0·97) | 0·010 |
| **Best/Worst Case Scenario II^d^** *(Baseline: experiencing bullying victimisation; Follow-up: considered as experiencing no bullying victimisation)* | *11,523* |  |  |
| Usual practice |  | Reference |  |
| KiVa |  | 0·86 (0·78, 0·95) | 0·004 |
| **Best/Worst Case Scenario III^d^**  *(Both Baseline and Follow-up considered as experiencing bullying victimisation)* | *11,523* |  |  |
| Usual practice |  | Reference |  |
| KiVa |  | 0·85 (0·78, 0·93) | 0·001 |
| **Best/Worst Case Scenario IV^d^**  *(Baseline: experiencing no bullying victimisation and Follow-up considered as experiencing bullying victimisation)* | *11,523* |  |  |
| Usual practice |  | Reference |  |
| KiVa |  | 0·86 (0·79, 0·93) | <0·001 |

*OR=Odds ratio; CI=confidence interval. OR>1 indicates a higher rate of bullying in KiVa arm compared to UP; OR<1 indicates a higher rate of bullying in usual practice arm compared to KiVa. a adjusted for its corresponding baseline measures, FSM, KS2 size, age and gender of pupil and clustered within 118 schools within 4 recruitment sites; b complete baseline and 12 month follow-up data; c The model above is based on 6,482 responses, adjusted for FSM, KS2 size, age and gender of pupil and clustered within 38 schools within 4 recruitment sites; d The model above is based on 11,523 responses, adjusted for FSM, KS2 size, age and gender of pupil and clustered within 118 schools within 4 recruitment sites.*

### **Subgroup analyses**

Table S2·1 presents the subgroup analyses for primary outcome. Two subgroups were pre-specified: Pupil gender (boy/girl) and the proportion of pupils eligible for Free School Meals (low/high). Three additional subgroups were performed: Pupils self-reported age (6-7 years, 8 years, 9 years, 10/11 years old), whether the school was previously recruited before COVID-19 pandemic for Stand Together trial (not previously recruited/previously recruited) and the timing of baseline data collection (before or after the school had been informed of randomisation).

The number of girls and boys in subgroup analyses were based on 5,730 and 5,977 respectively; the rate of bullying for girls compared to boys at baseline in both KiVa and usual practice arm are similar. Though, at 12-month follow up, the rate of bullying increased overtime in both arms for girls but reduced in boys. Within strata effects were similar for boys and girls and there was no difference between the two (interaction p-value=0.733). Based on pupil’s age stratum, 6/7 years old pupils had increased rate of bullying victimisation at 12 month after intervention whereas in 10/11 years old the rate of bullying victimisation were declined. There was no subgroup effect of proportion of pupils age on bullying victimisation (p value=0·733).

The proportion of bullying victimisation at baseline and 12-month follow up are 20·0% and 18·1% in KiVa arm respectively when there is high FSM eligibility. Alternatively for low FSM eligible, in KiVa arm, the proportion of bullying victimisation at baseline and 12-month follow up are 20·5% and 17·4%. It shows that there was no subgroup effect of proportion of pupils FSM eligibility on bullying victimisation (p value=0·361).

A total of 57 schools out of 114 schools had pupils completing baseline data in 2020 prior to COVID 19 pandemic. In these, 47 schools were re-recruited and 10 were not recruited for the participation in post COVID study. This information was used to create a flag to indicate the schools included in the original data collection and included an interaction between trial arm and flag in the main primary outcome model. This shows the sensitivity analysis on the effect of previous participation. This indicated that schools that did not participate prior to the Covid-19 pandemic (N=47) observed an intervention effect KiVa (OR: 0·77, 95% CI: 0·63, 0·95) whereas schools that had not participated prior to the Covid-19 pandemic (N=71) observed no intervention effect KiVa. However, the test of interaction showed that there is no significant effect of previous participation on intervention effect (p value=0·088).

**Table S2.1: Subgroup analyses on pupil self-reporting bullying victimization, n (%)**

|  | | **12-month follow-up** | | |  | | |  | | |
| --- | --- | --- | --- | --- | --- | --- | --- | --- | --- | --- |
|  |  | **KiVa​** | **Usual practice** | **​Adjusted* OR (95% CI)​** | | | **Interaction ​**  **p-value** | | |  |
|  |  | **N=5,584** | **N=4,862** |  |  |  |  |  |  |  |
| **Pupil self-reported gender** | Boy | 414 (16·0) | 395 (18·3) | 0·91 (0·74, 1·12) | | 0·61 | | |  |  |
|  | Girl | 466 (19·6) | 476 (23·2) | 0·83 (0·67, 1·02) | |  |  |  |  |  |
| **Proportion of pupils eligible for free school meals** | High FSM | 408 (18·1)​ | 406 (21·1)​ | 0·90 ​ (0·73, 1·10)​ | | 0·73  ​ | | |  |  |
|  | Low FSM | 477 (17·4)​ | 467 (20·3)​ | 0·85 ​ (0·67, 1·07)​ | |  |  |  |  |  |
| **Pupil self-reported age** | 6/7 years | 122 (21·5) | 118 (27·3) | 0·86 (0·57, 1·29) | | 0·73 | | |  |  |
|  | 8 years | 308 (19·5) | 317 (23·4) | 0·86 (0·67, 1·10) | |  |  |  |  |  |
|  | 9 years | 277 (17·0) | 282 (19·7) | 0·82 (0·66, 1·02) | |  |  |  |  |  |
|  | 10/11 years | 173 (14·4) | 154 (15·6) | 0·99 (0·70, 1·39) | |  |  |  |  |  |
| **Recruited before COVID-19 pandemic for Stand Together trial (pre-post covid)** | Previously recruited^‡^ | 286 (16·9) | 254 (17·1) | 1·10 (0·81, 1·50) | | 0·09 | | |  |  |
|  | Not previously recruited | 599 (18·2) | 619 (22·6) | 0·77 (0·63, 0·95) | |  |  |  |  |  |
| **Timing of baseline data collection** | Before school informed of randomisation | 353 (19·6) | 337 (20·2) | | 0·90 (0·71, 1·15) | | | 0·19 | | |
|  | After school informed of randomisation | 492 (16·6) | 501 (21·0) | | 0·84 (0·68, 1·04) | | |  | | |

CI=confidence interval; OR=odds ratio;

* Adjusted for its corresponding baseline measure, FSM, KS2 size, age and gender of pupil and clustered within 118 schools within 4 recruitment sites

‡ Recruited for Stand Together Trial in October-December 2019 (pre-COVID) - baseline data collected pre-COVID (not used for trial), then re-collected for the main trial in 2020.

### **Teacher secondary outcomes**

**Table S3.1: Secondary outcomes for teachers (Data source: Teacher questionnaire)**

|  | **12-month follow-up** | |  |  |  |
| --- | --- | --- | --- | --- | --- |
|  | **KiVa** | **Usual practice** | **Adjusted* difference in means effect (95% CI)** | **p value** | **Standardized effect size**† **(95% CI)** |
| **Total Teachers** | **N=220** | **N=226** | .. | .. |  |
| **MBI-GS**Median (IQR) ​ |  |  |  |  |  |
| Emotional exhaustion | 15 (10, 22) | 15 (9, 22) | -0·01^‡^ (-1·09, 1·06) | 0·98 | 0·04 (-0·15, 0·23) |
| Cynicism | 7 (2.5, 13.5) | 8 (3, 14) | -0·26^‡^ (-0·76, 0·23) | 0·29 | -0·1 (-0·29, 0·09) |
| Professional efficacy | 29 (25, 32) | 29 (25, 33) | -172·9^‡^ (-442·9, 97·1) | 0·21 | -0·11 (-0·30, 0·08) |
| **WEMWBS** Mean (SD) | 50.3 (8·8) | 50.1 (8·3) | 0·04 (-2·46, 2·55) | 0·98 | 0·02 (-0·16, 0·21) |
| **CBSES** Mean (SD) | 28 (25, 31) | 28 (24, 30) | 21·29^‡^ (-3·80, 46·4) | 0·16 | 0·18 (-0·01, 0·37) |

Data are n (%). Mean (SD) or median (IQR). MBI-GS= Maslach Burn-Out Inventory General Survey. WEMWBS=Warwick-Edinburgh Mental Well-being Scale. CBSES=Challenging Behaviour Self-Efficacy Scale. *Adjusted for stratification (site, free school meals KS2 school size), and clustered within 118 schools within 4 recruitment sites. † Glass’s delta – Standardised effect size calculated as the difference in means (KiVa - UP) divided by the standard deviation of the Usual Practice group at 12-month follow-up. ‡ Outcome measure transformed using Box Cos transformation to fulfil regression assumptions. Adjusted difference in means for KiVa minus usual practice presented in transformed value.

3.2 Exploratory analyses: Strengths and Difficulties Questionnaire (Teacher reported)

**Table S3.2:** Removing items on bullying from Strengths and Difficulties Questionnaire

**(Data source: Teacher questionnaire)**

|  | **12-month follow-up** | |  |  |
| --- | --- | --- | --- | --- |
|  | **KiVa** | **Usual practice** | **Adjusted* intervention effect (95% CI)** | **p value** |

| *** Conduct problems score** |  |  |  |  |
| --- | --- | --- | --- | --- |
| Normal | 5,356 (96.3) | 4,844 (96.3) |  |  |
| Borderline | 107 (1.9) | 97 (1.9) | reference |  |
| Abnormal | 98 (1.8) | 90 (1.8) | 0.99 (0.67, 1.47) | 0.975 |
| *** Peer problem score** |  |  |  |  |
| Normal | 4,853 (87.3) | 4,321 (85.9) |  |  |
| Borderline | 343 (6.2) | 372 (7.4) | reference |  |
| Abnormal | 364 (6.6) | 338 (6.7) | 0.93 (0.77, 1.13) | 0.455 |

**^*^Questionnaire items removed:**

**“**Often fights with other children or bullies them (removed from conduct problem scale)

and

“Picked on or bullied by other children” (removed from peer problem scale)

### **Exploratory analysis: Mediation analyses**

Figures 4.1 and 4.2 present the schematic representation of meditation analysis to find the effectiveness of intervention on bullying victimisation with change in affective empathy and change self-efficacy in defending respectively. Table S4.1 presents the direct, indirect and total effect of intervention and corresponding mediator on bullying victimisation. These models indicated that as compared to usual practice, 22% effect of KiVa is mediated through change in affective empathy whereas 12.5% of this effect mediated through change in self-efficacy in defending.

**Figure 4.1 The effect of intervention in bullying victimisation with changes in affective empathy**

**
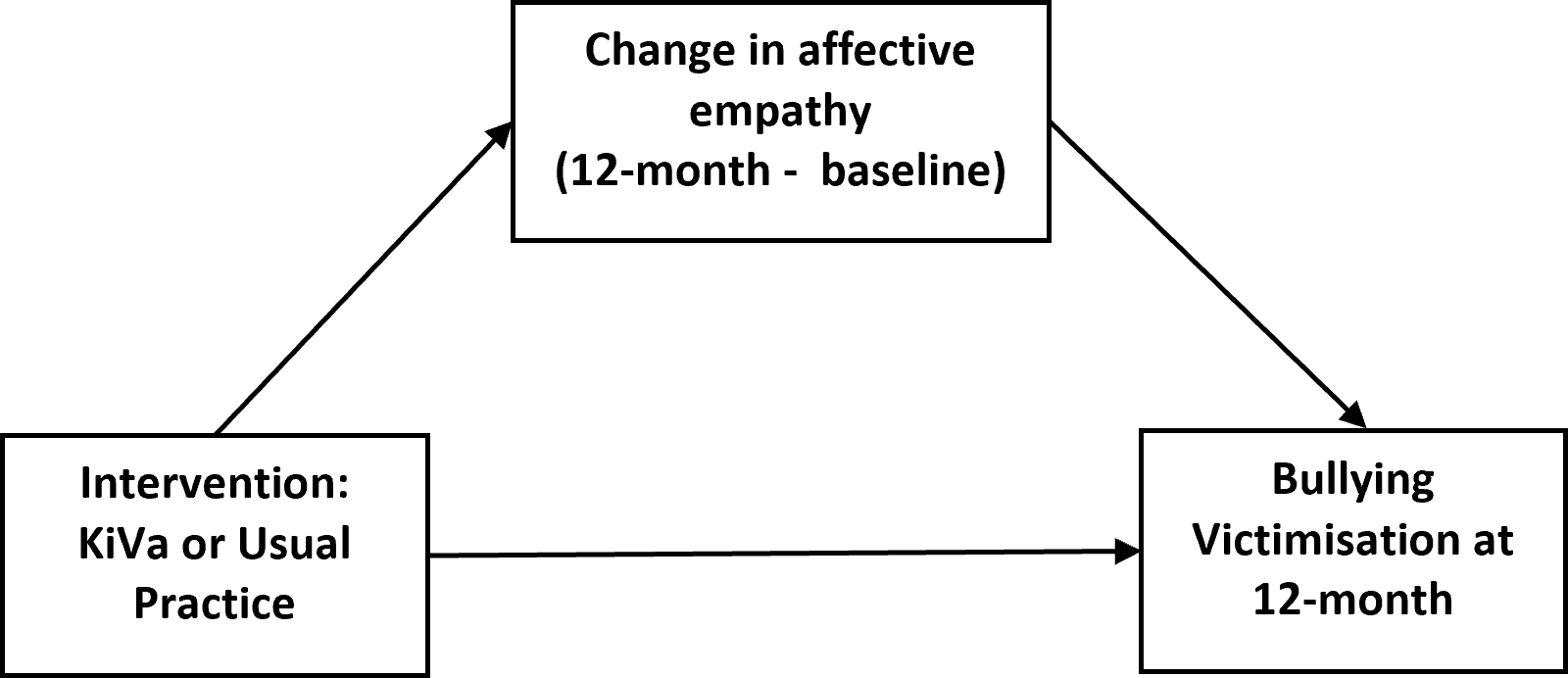
**

**Figure 4.2: The effect of intervention in changes in self-efficacy in defending bullying victimisation**

**
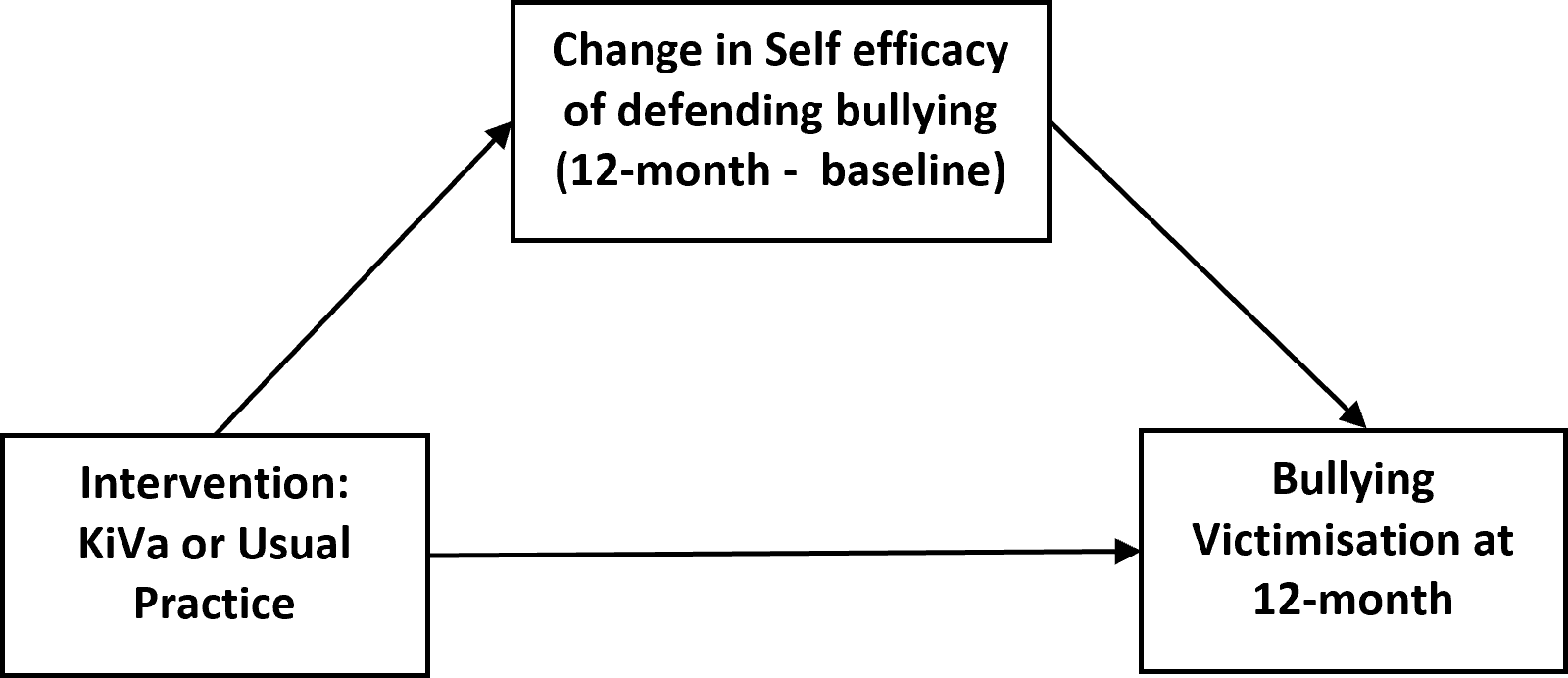
**

**Table S4.1**

| **Bullying Victimisation^a^** | **Direct Effects (a)** | | **Total Effects (b)** | | **Indirect Effects**  **(c) = (b)-(a)** | **Mediation effect in %**  **\|(c)/(b)\|^a^100** |
| --- | --- | --- | --- | --- | --- | --- |
|  | **Coefficient**  **(95% CI)** | **P value** | **Coefficient**  **(95% CI)** | **P value** |  |  |
| **Mediation through empathy** |  |  |  |  |  |  |
| KiVa | -0.14 (-0.25, -0.04) | 0.009 | -0.18 (-0.31, -0.05) | 0.005 | -0.04 | 22.2% |
| **Mediation through defending bully** |  |  |  |  |  |  |
| KiVa | -0.14 (-0.25, -0.04) | 0.009 | -0.16 (-0.28, -0.04) | 0.011 | -0.02 | 12.5% |

a The model is adjusted for its baseline measures, FSM, KS2 size, age and gender of pupil and clustered within 118 schools within 4 recruitment sites.

### **Resource use and costs**

**Table S5.1. KiVa programme cost components and unit costs**

| **Cost component** | **Description and assumptions** | **Unit cost** | **Sources** |
| --- | --- | --- | --- |
| ***YEAR 1*** | | | |
| ***Non-recurrent (training and set-up) costs*** | | | |
| KiVa training fee | Two-day (12 hour) training for two school staff members, delivered live or online. Online training is generally split into four half-days. | £600+VAT per school | Children’s Early Intervention Trust (CEIT) |
| School staff time - *Attending KiVa training* | Two members of school staff (school-coordinators and KiVa Team leads) attend the 12-hour KiVa training. Based on the job roles of school coordinator and KiVa Team leads reported in checklists, it is assumed that at least one member of staff will be a Leadership teacher and the other member of staff a class teacher.    Unit costs for staff time are expressed in £ per minute and are based on national average salary for primary school staff in England (Class teacher = £37,498 per annum, Leadership teacher = £52,819 per annum (2021-2022)). Salary calculations assume an average 40-hour working week, 39-working weeks per annum, and include estimated employers’ on-costs (25%) on-costs of national insurance, pensions, and allowances. | Class teacher £0·50 per minute  Leadership teacher £0·71 per minute | [School workforce in England, Reporting year 2021 – Explore education statistics – GOV.UK (explore-education-statistics.service.gov.uk)](https://explore-education-statistics.service.gov.uk/find-statistics/school-workforce-in-england/2021) |
| KiVa manual | The KiVa manual provides background information, full implementation instructions, KiVa curriculum lesson plans, scripts for addressing highlighted bullying incidents, and forms for dealing with incidents.  1 manual per KS2 teacher is required. | £50 per manual | CEIT |
| KiVa resource pack | Each resource pack contains 6 KiVa posters and 4 KiVa vests for school staff. 1 resource pack per 200 KS2 pupils is recommended by CEIT. | £37·50+VAT per resource pack | CEIT |
| ***YEAR 1*** | | | |
| ***Recurrent costs*** | | | |
| Annual school registration fee in first year. Includes access to online resources to support programme delivery | *School size => 50 KS2 pupils* | £2·50+VAT per pupil  (Min. charge £200+VAT per school) | CEIT |
|  | *School size < 50 KS2 pupils* | £2·50+VAT per pupil + £50+VAT | CEIT |
| School coordinator time – *Setting-up and coordinating KiVa* | School coordinator time spent setting-up and coordinating KiVa in first year of KiVa programme delivery.    School coordinator salary calculations are based on the job roles reported in school coordinator checklists and corresponding national average salary estimates for primary school staff in England (Class teacher = £37,498 per annum, Leadership teacher = £52,819 per annum, Headteacher = £68,745 per annum (2021-2022)). Salary calculations assume an average 40-hour working week, 39-working weeks per annum, and include estimated employers’ on-costs (25%) on-costs of national insurance, pensions, and allowances. Unit costs for school coordinator time are expressed in £ per minute. | Teaching assistant £0·23 per minute  Class teacher £0·50 per minute  Leadership teacher £0·71 per minute  Headteacher £0·92 per minute | [School workforce in England, Reporting year 2021 – Explore education statistics – GOV.UK (explore-education-statistics.service.gov.uk)](https://explore-education-statistics.service.gov.uk/find-statistics/school-workforce-in-england/2021) |
| Teaching assistant time – *Facilitating KiVa online pupil survey completion* | Teaching assistant time spent facilitating pupil completion of online KiVa survey each year, based on an estimate of 83 minutes per class reported by Clarkson et al (2019).    Teaching assistant salary calculations are based on national average mid-point salary estimates provided by the National Careers Service UK (Teaching assistant = £17,500 per annum (2022-2023)) and assume an average 40-hour working week, 39-working weeks per annum, and include estimated employers’ on-costs (25%) on-costs of national insurance, pensions, and allowances. Unit costs for teaching assistant time are expressed in £ per minute. | Teaching assistant £0·23 per minute | [Teaching assistant \| Explore careers \| National Careers Service](https://nationalcareers.service.gov.uk/job-profiles/teaching-assistant) |
| Class teacher time – *Preparing and delivering KiVa lessons* | Class teacher time spent preparing and delivering KiVa lessons.    Class teacher salary calculations are based on national average salary estimates for primary school staff in England (Class teacher = £37,498 per annum (2022-2023)) and assume an average 40-hour working week, 39-working weeks per annum, and include estimated employers’ on-costs (25%) on-costs of national insurance, pensions, and allowances. Unit costs for teaching assistant time are expressed in £ per minute. |  | [School workforce in England, Reporting year 2021 – Explore education statistics – GOV.UK (explore-education-statistics.service.gov.uk)](https://explore-education-statistics.service.gov.uk/find-statistics/school-workforce-in-england/2021) |
| UK-wide KiVa trainer support from CEIT | On-going implementation support from a CEIT-employed KiVa trainer is available to all KiVa-registered schools in the UK. | £7,500 per annum across 164 schools | CEIT |
| UK-wide KiVa administrative support from CEIT | On-going administrative support from a CEIT-employed administrator is available to all KiVa-registered schools in the UK. Estimated average weekly administrator time spent supporting KiVa-registered schools is 0·5 days (3·75 hours) per week.    The administrator salary cost calculation assumes an average 37·5-hour working week, 44-working weeks per annum, and includes estimated employers’ on-costs (25%) on-costs of national insurance, pensions, and allowances. | Administrator £0·29 per minute | Bangor University pay scales and rates |
| ***Year 2*** | | | |
| ***Recurrent costs*** | | | |
| Annual school registration fee in subsequent years. Includes access to online resources to support programme delivery | *School size => 50 KS2 pupils* | £2·00+VAT per pupil  (Min· charge £150+VAT per school) | CEIT |
|  | *School size < 50 KS2 pupils* | £2·00+VAT per pupil + £50+VAT | CEIT |
| School coordinator time – *On-going coordination of KiVa* | School coordinator time spent coordinating the delivery of KiVa in school. On-going annual school coordinator activities include:   - Re-launching programme and informing parents - Monitoring KiVa lesson delivery - Training new staff as required - Co-ordinating KiVa pupil survey and disseminating results - Refining action plan for KiVa delivery in subsequent years     Time spent on the above activities is estimated by adjusting school coordinator time spent setting-up and coordinating KiVa during first year based on CEIT assumptions, informed by their experience of working with existing KiVa schools:  1^st^ term = Year 1 school coordinator time*0·5  2^nd^ term = Year 1 school coordinator time*0·66  3rd term = Year 1 school coordinator time*1    School coordinator salary calculations are based on the job roles reported in school coordinator checklists and corresponding national average salary estimates for primary school staff in England (Class teacher = £37,498 per annum, Leadership teacher = £52,819 per annum, Headteacher = £68,745 per annum (2021-2022)). Salary calculations assume an average 40-hour working week, 39-working weeks per annum, and include estimated employers’ on-costs (25%) on-costs of national insurance, pensions, and allowances. Unit costs for school coordinator time are expressed in £ per minute. | Teaching assistant £0·23 per minute  Class teacher £0·50 per minute  Leadership teacher £0·71 per minute  Headteacher £0·92 per minute | [School workforce in England, Reporting year 2021 – Explore education statistics – GOV.UK (explore-education-statistics.service.gov.uk)](https://explore-education-statistics.service.gov.uk/find-statistics/school-workforce-in-england/2021) |
| Teaching assistant time – *Facilitating KiVa online pupil survey completion* | Teaching assistant time spent facilitating pupil completion of online KiVa survey each year, based on an estimate of 83 minutes per class reported by Clarkson et al (2019).    Teaching assistant salary calculations are based on national average mid-point salary estimates provided by the National Careers Service UK (Teaching assistant = £17,500 per annum (2022-2023)) and assume an average 40-hour working week, 39-working weeks per annum, and include estimated employers’ on-costs (25%) on-costs of national insurance, pensions, and allowances. Unit costs for teaching assistant time are expressed in £ per minute. | Teaching assistant £0·23 per minute | [Teaching assistant \| Explore careers \| National Careers Service](https://nationalcareers.service.gov.uk/job-profiles/teaching-assistant) |
| Class teacher time – *Preparing and delivering KiVa lessons* | Class teacher time spent preparing and delivering KiVa lessons.    Class teacher salary calculations are based on national average salary estimates for primary school staff in England (Class teacher = £37,498 per annum (2022-2023)) and assume an average 40-hour working week, 39-working weeks per annum, and include estimated employers’ on-costs (25%) on-costs of national insurance, pensions, and allowances. Unit costs for teaching assistant time are expressed in £ per minute. | Class teacher £0·50 per minute | [School workforce in England, Reporting year 2021 – Explore education statistics – GOV.UK (explore-education-statistics.service.gov.uk)](https://explore-education-statistics.service.gov.uk/find-statistics/school-workforce-in-england/2021) |
| UK-wide KiVa trainer support from CEIT | On-going implementation support from a CEIT-employed KiVa trainer is available to all KiVa-registered schools in the UK. | £7,500 per annum across 164 schools | CEIT |
| UK-wide KiVa administrative support from CEIT | On-going administrative support from a CEIT-employed administrator is available to all KiVa-registered schools in the UK. Estimated average weekly administrator time spent supporting KiVa-registered schools is 0·5 days (3·75 hours) per week.    The administrator salary cost calculation assumes an average 37·5-hour working week, 44-working weeks per annum, and includes estimated employers’ on-costs (25%) on-costs of national insurance, pensions, and allowances. | Administrator £0·30 per minute | Bangor University pay scales and rates |

**Table S5.2. Average class teacher time spent on KiVa and PSHE/ PSE lesson preparation and delivery.**

| **School staff time (minutes)** | **N** | **Control,**  **Mean (SD)** | N | **Intervention,**  **Mean (SD)** |
| --- | --- | --- | --- | --- |
| Preparation time per KiVa lesson | - | - | 38 | 25·29 (12·62) |
| Delivery time per KiVa lesson | - | - | 38 | 70·93 (40·43) |
| Preparation time per PSHE/PSE lesson | 44 | 26·90 (16·18) | - | - |
| Delivery time per PSHE/PSE lesson | 43 | 51·64 (19·88) | - | - |

**Table S5.3. Costs per pupil associated with the KiVa intervention and usual practice, estimated for the first year of KiVa intervention delivery and subsequent years.**

| **Costs (£)** | | **N** | **Control,**  **Mean (SD)** | **N** | **Intervention,**  **Mean (SD)** |  |
| --- | --- | --- | --- | --- | --- | --- |
| ***Year 1*** | | | | | | |
| **Non-recurrent costs** | KiVa training (training fee + school staff time spent training) | - | - | 59 | 15·15 (9·58) |  |
|  | KiVa manuals and resource packs | - | - | 59 | 2·33 (0·48) |  |
| **Recurrent costs** | KiVa registration fee | - | - | 59 | 2·77 (0·45) |  |
|  | Setting-up and coordinating KiVa (School coordinator) - Term 1 | - | - | 30 | 1·45 (1·10) |  |
|  | Setting-up and coordinating KiVa (School coordinator) - Term 2 | - | - | 27 | 1·03 (0·85) |  |
|  | Setting-up and coordinating KiVa (School coordinator) - Term 3 | - | - | 14 | 1·16 (1·22) |  |
|  | Facilitating KiVa pupil survey completion (Teaching assistant) | - | - | 59 | 0·73 (0·12) |  |
|  | Preparing KiVa lessons (Class teacher) | - | - | 38 | 3·62 (1·74) |  |
|  | Delivering KiVa lessons (Class teacher) | - | - | 38 | 9·57 (4·14) |  |
|  | UK-wide support from CEIT  (Includes KiVa trainer and admin support) | - | - | 59 | 0·36 |  |
|  | Preparing 40% PSHE/PSE lessons (Class teacher) | 44 | 6·05 (3·41) | - | - |  |
|  | Delivering 40% PSHE/PSE lessons (Class teacher) | 43 | 11·34 (4·99) | - | - |  |
| **Total Year 1 costs** | | **59** | **17.40 (6.74)** | **59** | **38·18 (11·36)** |  |
| ***Subsequent years*** | | | | | | |
| **Recurrent costs** | KiVa registration fee |  | - | 59 | 2·20 (0·38) |  |
|  | Setting-up and coordinating KiVa (School coordinator) - Term 1 | - | - | 30 | 0·72 (0·55) |  |
|  | Setting-up and coordinating KiVa (School coordinator) - Term 2 | - | - | 27 | 0·68 (0·56) |  |
|  | Setting-up and coordinating KiVa (School coordinator) - Term 3 | - | - | 14 | 1·16 (1·22) |  |
|  | Facilitating KiVa pupil survey completion |  | - | 59 | 0·73 (0·12) |  |
|  | Preparing KiVa lessons (Class teacher) |  | - | 38 | 3·62 (1·74) |  |
|  | Delivering KiVa lessons (Class teacher) |  | - | 38 | 9·57 (4·14) |  |
|  | UK-wide support from CEIT  (Includes KiVa trainer and admin support) | - | - | 59 | 0·36 |  |
|  | Preparing 40% PSHE/PSE lessons (Class teacher) | 44 | 6·05 (3·41) | - | - |  |
|  | Delivering 40% PSHE/PSE lessons (Class teacher) | 43 | 11·34 (4·99) | - | - |  |
| **Total mean subsequent year costs** | | **59** | **17·40 (6·74)** | **59** | **19·05 (3·94)** |  |

**Table S5**.**4. Costs per pupil associated with the KiVa intervention and usual practice, estimated for the first year of KiVa intervention delivery and subsequent years.**

| **Costs (£)** | | **N** | **Control,**  **Mean (SD)** | **N** | **Intervention,**  **Mean (SD)** |
| --- | --- | --- | --- | --- | --- |
| ***Year 1*** | | | | | |
| **Non-recurrent costs** | KiVa training (training fee + school staff time spent training) | - | - | 59 | 15·15 (9·58) |
|  | KiVa manuals and resource packs | - | - | 59 | 2·33 (0·48) |
| **Recurrent costs** | KiVa registration fee | - | - | 59 | 2·77 (0·45) |
|  | Setting-up and coordinating KiVa (School coordinator) - Term 1 | - | - | 30 | 1·45 (1·10) |
|  | Setting-up and coordinating KiVa (School coordinator) - Term 2 | - | - | 27 | 1·03 (0·85) |
|  | Setting-up and coordinating KiVa (School coordinator) - Term 3 | - | - | 14 | 1·16 (1·22) |
|  | Facilitating KiVa pupil survey completion (Teaching assistant) | - | - | 59 | 0·73 (0·12) |
|  | Preparing KiVa lessons (Class teacher) | - | - | 38 | 3·62 (1·74) |
|  | Delivering KiVa lessons (Class teacher) | - | - | 38 | 9·57 (4·14) |
|  | UK-wide support from CEIT  (Includes KiVa trainer and admin support) | - | - | 59 | 0·36 |
|  | Preparing 40% PSHE/PSE lessons (Class teacher) | 44 | 6·05 (3·41) | - | - |
|  | Delivering 40% PSHE/PSE lessons (Class teacher) | 43 | 11·34 (4·99) | - | - |
| **Total Year 1 costs** | | **59** | **17·40 (6·74)** | **59** | **38·18 (11·36)** |
| ***Subsequent years*** | | | | | |
| **Recurrent costs** | KiVa registration fee |  | - | 59 | 2·20 (0·38) |
|  | Setting-up and coordinating KiVa (School coordinator) - Term 1 | - | - | 30 | 0·72 (0·55) |
|  | Setting-up and coordinating KiVa (School coordinator) - Term 2 | - | - | 27 | 0·68 (0·56) |
|  | Setting-up and coordinating KiVa (School coordinator) - Term 3 | - | - | 14 | 1·16 (1·22) |
|  | Facilitating KiVa pupil survey completion |  | - | 59 | 0·73 (0·12) |
|  | Preparing KiVa lessons (Class teacher) |  | - | 38 | 3·62 (1·74) |
|  | Delivering KiVa lessons (Class teacher) |  | - | 38 | 9·57 (4·14) |
|  | UK-wide support from CEIT  (Includes KiVa trainer and admin support) | - | - | 59 | 0·36 |
|  | Preparing 40% PSHE/PSE lessons (Class teacher) | 44 | 6·05 (3·41) | - | - |
|  | Delivering 40% PSHE/PSE lessons (Class teacher) | 43 | 11·34 (4·99) | - | - |
| **Total subsequent year costs** | | **59** | **17·40 (6·74)** | **59** | **19·05 (3·94)** |

1. White IP, Royston P, Wood AM. Multiple imputation using chained equations: issues and guidance for practice. Stat Med 2011; 30:377.e46522-377.e46599 [↑](#footnote-ref-1)
